# Qihuang Granule protects the retinal pigment epithelium from oxidative stress via regulation of the alternative complement pathway

**DOI:** 10.1186/s12906-023-03884-2

**Published:** 2023-02-18

**Authors:** Yan Wang, Huangxuan Shen, Long Pang, Bo Qiu, Yuan Yuan, Xiaoying Guan, Xiaolan Xiang

**Affiliations:** 1 Department of Ophthalmology, The Second Affiliated Hospital of Guangzhou University of Chinese Medicine, 111 Dade Road, Guangzhou, 510120 China; 2 State Key Laboratory of Ophthalmology, Zhongshan Ophthalmic Center, Sun Yat-sen University, 54 Xianlie Road, Guangzhou, 510060 China; 3 The Second Clinical college of Guangzhou University of Chinese Medicine, Guangzhou, 510006 China

**Keywords:** Age-related macular degeneration, Alternative complement pathway, Oxidative stress, Traditional Chinese medicine, Qihuang Granule

## Abstract

**Background:**

Age-related macular degeneration (AMD) is a leading cause of vision loss in elderly people, and dry AMD is the most common type of AMD. Oxidative stress and alternative complement pathway activation may play essential roles in the pathogenesis of dry AMD. There are no available drugs for dry AMD. Qihuang Granule (QHG) is an herbal formula for the treatment of dry AMD, and it achieves a good clinical effect in our hospital. However, its potential mechanism is unclear. Our study investigated the effects of QHG on oxidative stress-associated retinal damage to reveal its underlying mechanism.

**Methods:**

Oxidative stress models were established using H_2_O_2_ and NaIO_3_ in ARPE-19 cells and C57BL/6 mice. Cell apoptosis and viability were assessed using phase contrast microscopy and flow cytometry, respectively. Alterations in the mouse retinal structure were evaluated using Masson staining and transmission electron microscopy (TEM). The expression of complement factor H (CFH), complement component 3a (C3a) and complement component 5a (C5a) in retinal pigment epithelium (RPE) cells and mice was measured using RT‒PCR, Western blot analysis and ELISA.

**Results:**

Pretreatment with QHG significantly prevented cell apoptosis and disorder of the RPE and inner segment/outer segment (IS/OS) in H_2_O_2_-treated RPE cells and NaIO_3_-injected mice. QHG alleviated mitochondrial damage in mouse RPE cells, as shown by TEM. QHG also promoted CFH expression and inhibited the expression of C3a and C5a.

**Conclusions:**

The results suggest that QHG protects the retinal pigment epithelium from oxidative stress, likely by regulating the alternative complement pathway.

**Supplementary Information:**

The online version contains supplementary material available at 10.1186/s12906-023-03884-2.

## Background

Age-related macular degeneration (AMD) is a leading cause of irreversible vision loss in elderly people, and it accounts for 8.7% of cases of blindness worldwide. It is projected that the total number of patients with AMD in 2040 will be as high as 288 million globally [1]. There are two forms of AMD: dry AMD and wet AMD. Dry AMD is the most common type of AMD, and it accounts for approximately 80-90% of AMD patients [2]. Dry AMD is characterized by progressive degeneration of the retinal pigment epithelium (RPE) and photoreceptor cells [3]. Geographic atrophy, which is the late stage of dry AMD, leads to severe vision loss [4, 5]. There are no effective prevention strategies or drugs available for the treatment of dry AMD, so treatment remains a challenge [6].

Although the mechanisms of dry AMD are not known, accumulating studies have shown that oxidative stress-induced RPE damage may play an essential role in the pathogenesis of dry AMD [7]. The RPE consumes high levels of oxygen, and it is specifically sensitive to oxidative stress [8]. Oxidative stress produces retinal reactive oxygen species (ROS), which induce the formation of mitochondrial DNA lesions and programmed necrosis in RPE cells [9]. H_2_O_2_ and NaIO_3_ are well-known toxic oxidative reagents that generate ROS and selectively damage RPE cells. These agents are often used to establish an oxidative stress model of AMD [10].

In addition to oxidative stress-induced RPE damage, alternative complement pathway activation, which always occurs simultaneously with oxidative stress, is a major cause of dry AMD [11]. Immunohistological and proteomic analyses of human donor eyes from patients with AMD revealed the expression of a large number of complement components and activation products, including complement factor H (CFH), complement factor B (CFB), complement component 3a (C3a), and complement component 5a (C5a), in drusen [12]. Genetic studies strongly support a relationship between the development of AMD and the alternative complement cascade, particularly the common H402 variant in CFH [13]. CFH is a crucial inhibitor of the alternative complement pathway, and downregulation of CFH expression induces continuous activation of the alternative complement pathway [14]. C3a and C5a, which are the complement fragments of complement 3 (C3) and complement 5 (C5), are anaphylatoxins and proinflammatory stimuli that play important roles in local chronic retinal inflammation [15, 16].

Qihuang Granule (QHG) is a traditional Chinese medicine (TCM) herbal recipe that was prescribed by Professor Fengming Liang. QHG is commonly used in the clinical treatment of mild-to-moderate AMD in China [17]. The granule consists of *Salvia* *miltiorrhiza* Bunge, *Lycium barbarum* L., *Leonurus cardiaca* L., and *Broussonetia kaempferi* Siebold. Many studies have demonstrated that these single herbs have multiple pharmacological and biological functions, including neuroprotective [18], antioxidant [19], anti-inflammatory [19, 20], and immunomodulatory properties [21, 22], in various cells and tissues. Our previous study revealed that QHG improved the vision of AMD patients [17], reduced the apoptosis of RPE cells in mice subjected to light-induced retinal damage [23], and increased the serum CFH level and decreased the serum C5b9 level in AMD patients [24]. However, the effects of QHG on oxidative stress-induced retinal damage were not examined.

Thus, this study aimed to further investigate whether QHG protects the RPE by modulating the alternative complement pathway. We used H_2_O_2_ and NaIO_3_ to establish oxidative stress models in ARPE-19 cells and C57BL/6 mice, respectively. The cell and mouse models were treated with appropriate doses of QHG, and the related indexes of dry AMD were observed. We attempted to illustrate the intrinsic mechanism, and the results of the present study are helpful in understanding TCM prevention and the treatment of dry AMD and support the clinical efficacy of QHG.

## Methods

### Reagents

High glucose Dulbecco's modified Eagle's medium (DMEM; HyClone; GE Healthcare Life Sciences, Logan, UT, USA), foetal bovine serum (Thermo Fisher Scientific, Inc., Waltham, MA, USA), a MultiCaspase Assay Kit (Millipore, MA, USA), 7-aminoactinomycin D (7-AAD, Millipore, MA, USA), MTT (5 mg/ml; Sigma‒Aldrich and Merck KGaA, Darmstadt, Germany), dimethyl sulfoxide (DMSO), NaIO_3_ (Sigma‒Aldrich and Merck KGaA, Darmstadt, Germany), TRIzol Reagent (Takara, Japan), a PrimeScrip-tII 1st Strand cDNA Synthesis Kit (TaKaRa, Japan), a SYBR Premix Ex TaqTM Kit (TaKaRa, Japan), Protein Marker (Fermentas, Canada), PVDF membrane (Millipore, MA, USA), BeyoECL Plus (Tanon, Shanghai, China), Penicillin, Streptomycin, RIPA buffer, PMSF, Proteinase inhibitors, Protein loading buffer, an SDS‒PAGE gel preparation kit, and Skimmed milk powder (Servicebio, Wuhan, China) were used.

### Preparation of QHG

QHG comprises four herbs, as described in Table 1. All the mentioned herbs can be found in the database of www.theplantlist.org. The herbs were purchased from Xuzhou Pharmaceutical Co., Ltd. (Jiangsu, China). They were identified by Tang Bo, an associate professor major in Traditional Chinese Medicine. The voucher specimens were deposited in Jiangyin Tianjiang Pharmaceutical Co., Ltd. The batch number is 1612350. To prepare the QHG sample, the four dried herbs were crushed into crude grains and sieved through a 20 mesh sieve. Then, 200 g of QHG powder was placed in a flask, and 2000 ml of water was added, boiled and refluxed in the flask for 1 h and then filtered. The extraction process was repeated twice, and the combined filtrates were evaporated to 1.15-1.20 g/ml. For the cell experiments, QHG was dissolved in serum-free medium to a concentration of 200 mg/ml and stored at 4 °C for later use.

**Table 1:** Information on the components in the Qihuang Granule (QHG)

**Botanical name**	**Herbal name**	**Chinese name**	**Voucher No.**	**Ratio**
*Salvia miltiorrhiza* Bunge	Radix salvia miltiorrhiza	Dan Shen	SCM20170631	2
*Lycium barbarum* L.	Fruit lycium barbarum	Gou Qi	SCM20177696	1
*Leonurus cardiaca* L.	Fructus leonuri	Chong Wei Zi	SCM20173782	1
*Broussonetia kaempferi* Siebold.	Fructus broussonetiae	Zhu Shi Zi	SCM20172066	1

### Cell culture

The human RPE cell line ARPE-19 was obtained from the American Type Culture Collection (Manassas, VA, USA). The cells were cultured in DMEM with 10% foetal bovine serum, penicillin (100 U/ml) and streptomycin (50 U/ml) in a 5% CO_2_ humidified environment at 37 °C.

### Cell apoptosis analysis

Apoptotic cells were identified based on morphological changes and the apoptotic rate using a phase-contrast microscope and flow cytometry. Briefly, ARPE-19 cells were seeded in a six-well plate at 1×10^4^ cells/well and pretreated with various concentrations of QHG for 24 h. Cells were treated with 200 μM H_2_O_2_ for an additional 24 h or were untreated. Cells were collected and incubated in multicaspase reagent for 30 min at 37 °C in the dark and then incubated with 7-AAD for 5 min at room temperature. Cell apoptosis was detected using a phase contrast microscope and flow cytometry. All procedures were performed according to the manufacturer's protocols, and flow cytometry was performed using a FACScan (Beckman Coulter, Fullerton, CA, USA).

### Cell viability assay

Cell viability was measured using the MTT assay. Briefly, ARPE-19 cells were seeded in 96-well plates at a density of 4,000 cells/well. The cells were cultured with different concentrations of QHG for 24, 48, and 72 h. Thiazolyl blue tetrazolium bromide (5 mg/ml) in PBS was added to each well and incubated for another 4 h at 37 ℃. The supernatants were discarded, and the crystal violet was dissolved in 150 µl of DMSO. The 96‐well plates were shaken at room temperature for 10 min, and the absorbance was measured at 450 nm on a microplate reader.

### Animals

Healthy male C57BL/6 mice (six weeks old, 20 ± 2 g) were purchased from the Experimental Animal Center of Guangzhou Traditional Medical University. The Experimental Animal Ethics Committee of Guangzhou University of Chinese Medicine approved the animal experiments (Approval number: 2017018). The experiments were performed in accordance with the Principles of the Care and Use of Laboratory Animals. All animals were raised in plastic cages (five mice in one cage) and housed in an environment at 22‐24 °C, relative humidity of 50 + 1% and a 12 h alternating light-dark cycle. Animals had free access to food and water. The animals were acclimated for at least 1 week before the experiments.

### Induction of experimental AMD

Sixty C57BL/6 mice were randomly divided into three groups (*n* = 20 for each group): the control group, model group, and QHG treatment group. The mice in the QHG treatment group were intragastrically administered QHG (at a dose of 1.17 g/kg/day, by oral gavage) for 7 days. Mice in the model and QHG treatment groups received NaIO_3_ (40 mg/kg) via the tail vein, as described in previous research. Mice in the control group were injected with an equal volume of 0.9% physiological saline via the tail vein. After the injection of NaIO_3_, mice in the QHG treatment group were continuously administered QHG, and mice in the control and model groups were intragastrically administered an equal volume of 0.9% physiological saline. On days 7, 14 and 28 after injection, 5 mice in each group were randomly removed, weighed and euthanized. Blood was collected from the abdominal aorta, and the eyes were harvested for follow-up experiments.

### Masson staining

The eyes were enucleated immediately after the mice were sacrificed, fixed in 4% paraformaldehyde and embedded in paraffin. Following routine procedures, 3 μm thick sections were obtained from the resulting paraffin blocks. After deparaffinization and rehydration, the paraffin sections were stained with Masson trichrome. All sections were examined under a light microscope (MSHOTML 31, Guangzhou, China).

### Transmission electron microscopy (TEM)

The eyes were enucleated and fixed in electron microscope fixative (G1102, Servicebio, Wuhan, China) for 4 h at 4 ℃. The anterior segments were removed under a stereomicroscope. Eyecups with the retina and choroid were fixed in 1% osmium tetroxide in 0.1 M phosphate buffer (pH 7.4) for 2 h at 20 ℃. The fixed eyecups were dehydrated in a gradient alcohol series and embedded in Epoxy EPON812. Ultrathin sections (70 nm) were cut using a Leica EM UC7 microtome and stained with uranyl acetate and lead citrate for 15 min. The stained specimens were analysed using a Hitachi HT7700 TEM instrument (Hitachi Co. Ltd., Tokyo, Japan).

### Reverse transcription quantitative polymerase chain reaction (RT‒PCR) analysis

Total RNA was extracted from ARPE-19 cells and the eyes of C57BL/6 mice using TRIzol reagent (Invitrogen, Carlsbad, CA, USA) and further purified using an RNA Micro Kit (Qiagen, Valencia, CA, USA). Total RNA was converted into cDNA using the PrimeScript II 1st Strand cDNA Synthesis kit (Takara Bio, Inc., Japan). RT‒PCR analysis was performed in a LightCycler 96 (Roche Diagnostics, Basel, Switzerland) using the SYBR Premix Ex TaqTM kit (Takara Bio, Inc., Japan). Primers for CFH, C3, and C5 were designed using Primer Blast and are listed in Table 2. The GAPDH gene served as the reference control. The relative mRNA expression levels were calculated using the comparative CT (2^-∆∆Ct^) scheme.

**Table 2:** Primers used for amplification of different markers

**Primer**	**Forward Primer (5-3′)**	**Reverse Primer (5-3′)**
Human CFH	TCATTGTTATGGTCCTTAGGAAA	GGAGTAGGAGACCAGCCATT
Murine CFH	TCATTGTTATGGTCCTTAGGAAA	TTAGAAAGACATGAACATGCTAGG
Human C3	AGAGGGCAGAACTTCAGTGC	CTTGGGGTACTTGCCGACTT
Murine C3	AGAGGGCAGAACTTCAGTGC	CTTGGGGTACTTGCCGACTT
Human C5	CGTTTCCCGCCTCTTTTGCA	CTGAAATGACATATCTGCAACGC
Murine C5	GGTACTGTTGGAAGGGACGC	TGACATATCTGCAACGCAATCC
Human GAPDH	CCTCAAGATCATCAGCAAT	CCATCCACAGTCTTCTGGGT
Murine GAPDH	CCCGCTTCGCTCTCTGCTCC	ACCAGGCGCCAATACGACC

### Western blot (WB) analysis

Total proteins from the retinas of mice were extracted using radioimmunoprecipitation assay (RIPA) lysis buffer (catalogue no. P0013B, Beyotime Biotechnology, Shanghai, China), and protein concentrations were measured using a bicinchoninic acid assay kit (catalogue no. P0010, Beyotime Biotechnology, Shanghai, China). Total protein (70 μg) was taken from each sample, and an appropriate volume of loading buffer was added. The solution was mixed, boiled for 10 min, and then stored at -80 °C. A total of 10 µg of the protein solution from each sample was loaded onto 10% gels, subjected to SDS‒PAGE, and transferred onto a polyvinylidene difluoride membrane. The membranes were blocked with 5% fat-free milk for 1.5 h at room temperature and incubated with CFH (bs-6949R; Bioss, MA, USA), C3a (21337-1-AP; Proteintech, Chicago, IL, USA) and C5a (bs-15197R; Beijing Biosynthesis Biotechnology Co., Ltd. Beijing, China) primary antibodies at 4 °C overnight. Membranes were washed three times with 1X TBST for 10 min. The membranes were incubated with secondary antibodies for 1 h and washed three times with 1X TBST for 10 min at room temperature. The membranes were added to a mixed ECL solution (ECLA and ECLB reagents) for 1-2 minutes on a double-layer transparent film. Exposure was started after the residual liquid was removed. Substrate (EMD Millipore) was added and detected using a Tanon 5200 Chemiluminescent Imaging System (Shanghai, China).

### Enzyme-linked immunosorbent assay (ELISA)

The concentrations of CFH, C3a, and C5a in the supernatant of ARPE-19 cells and mouse serum were measured using ELISA kits (CFH:CD10426; C3a:CD10421; C5a:CD10422; Wuhan Chundu Biotechnology Co., Ltd. Wuhan, China). All procedures were performed in strict accordance with the ELISA kit instructions.

### Statistical analysis

All experimental data were processed using SPSS 20.0 statistical software. The data were first tested for conformity to a normal distribution by a normal distribution test. If the data conformed to the normal distribution, the data were described using the description of the data, and if not, M(P25,P75) was used. Multiple data groups conforming to a normal distribution were analysed with chi-square tests or one-way ANOVA; data not conforming to a normal distribution were analysed using nonparametric tests. Differences were considered statistically significant when *p* < 0.05. Graphs were prepared using GraphPad Prism 8.0 software.

## Results

### QHG attenuated H_2_O_2_-induced RPE cell apoptosis

**Fig. 1:**
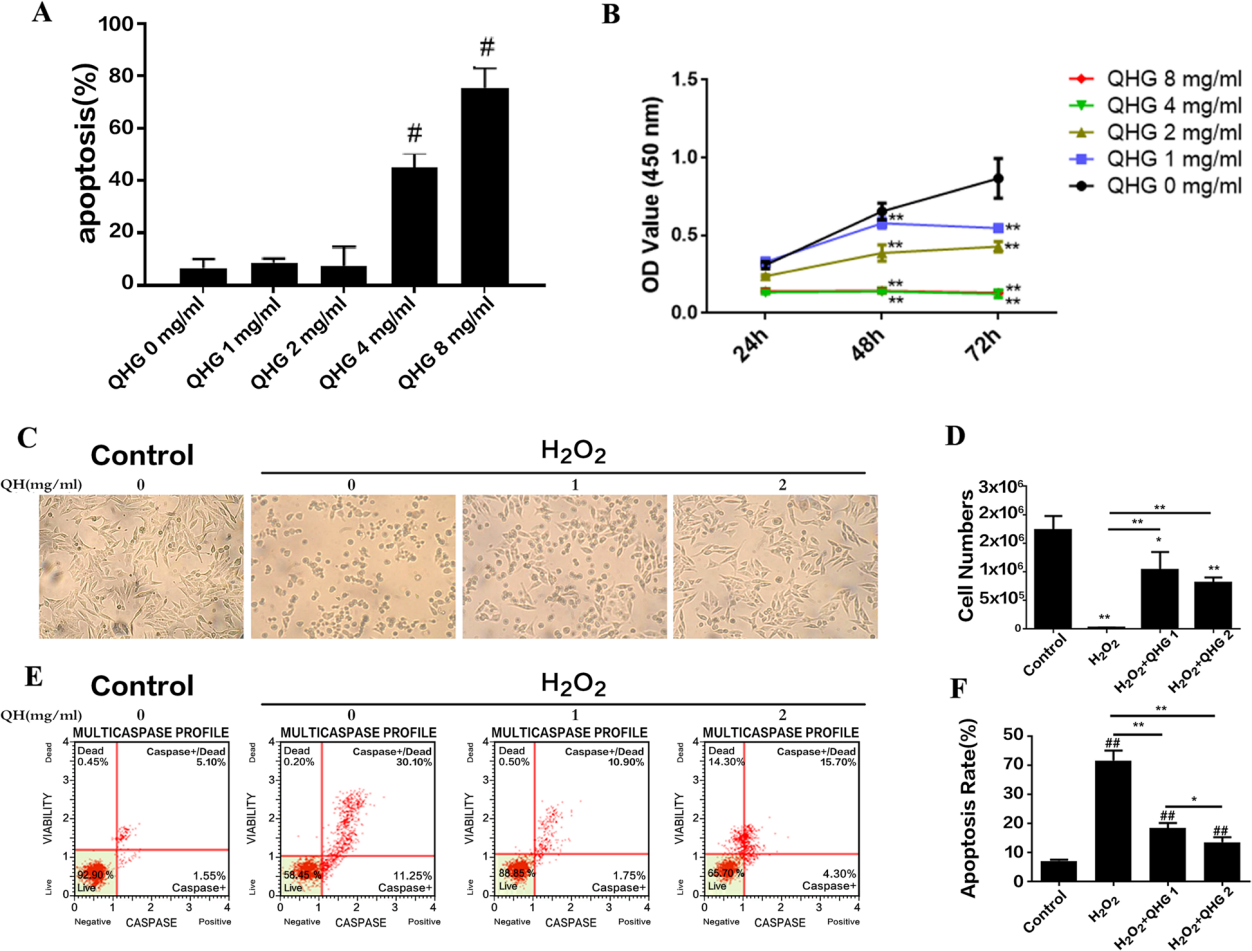
QHG attenuated H_2_O_2_-induced damage to RPE cells based on the MTT assay and flow cytometer analysis. **A** The cell apoptosis rate was evaluated without H_2_O_2_ treatment using flow cytometry. **B** Cell viability was measured without H_2_O_2_ treatment using the MTT assay. **C** ARPE-19 cells were pretreated with QHG (1 and 2 mg/ml) for 24 h and then exposed to 200 µM H_2_O_2_ for 24 h. Representative images of cell morphological changes were obtained from phase contrast microscopy. Scale bar, 100 μm. **D** The number of surviving ARPE-19 cells under a phase contrast microscope. **E**, **F** The apoptosis of ARPE-19 cells after pretreatment with QHG for 24 h followed by 200 µM H_2_O_2_ treatment for 24 h. All data were obtained from three independent experiments and are expressed as the means ± SDs. # *p* <0.05, ## *p* <0.01 vs. Control; *p < 0.05, **p < 0.01

To evaluate whether QHG protected ARPE-19 cells from H_2_O_2_-induced oxidative stress, the optimal concentration of QHG in ARPE-19 cells was determined first. ARPE-19 cells were treated with 1-8 mg/ml QHG for 24 h. The results showed that QHG at 4 mg/ml or more significantly increased apoptosis (Fig. 1A). Then, the optimal time of QHG treatment on ARPE-19 cells was evaluated. ARPE-19 cells were treated with 1-8 mg/ml QHG for 24 h, 48 h and 72 h. The survival rate of ARPE-19 cells cultured with 1 and 2 mg/ml QHG for 24 h was not significantly different (Fig. 1B). Therefore, a nontoxic dose of 2 mg/ml QHG for 24 h was used.

Next, ARPE-19 cells were incubated with 1 and 2 mg/ml QHG for 24 h and then treated with 200 μM H_2_O_2_ for the next 24 h. Phase contrast microscopy and flow cytometry were used to assess cell morphological changes and apoptosis levels. The ARPE-19 cells showed the typical cobblestone epithelial morphology in the control group, and they became round and shrunken in the H_2_O_2_-induced group. However, pretreatment with QHG significantly increased the number of normal ARPE-19 cells and restored normal morphology. The effect in the 2 mg/ml QHG group was more notable than that in the 1 mg/ml QHG group (Fig. 1C, D). Flow cytometry analysis also revealed that QHG pretreatment significantly decreased the apoptosis rates of ARPE-19 cells. Pretreatment with 2 mg/ml QHG had better antiapoptotic effects than pretreatment with 1 mg/ml QHG (Fig. 1E, F). These results suggested that QHG protected ARPE-19 cells from H_2_O_2_-induced cell apoptosis.

### QHG protected the retina from NaIO_3_-induced degeneration in mice

**Fig. 2:**
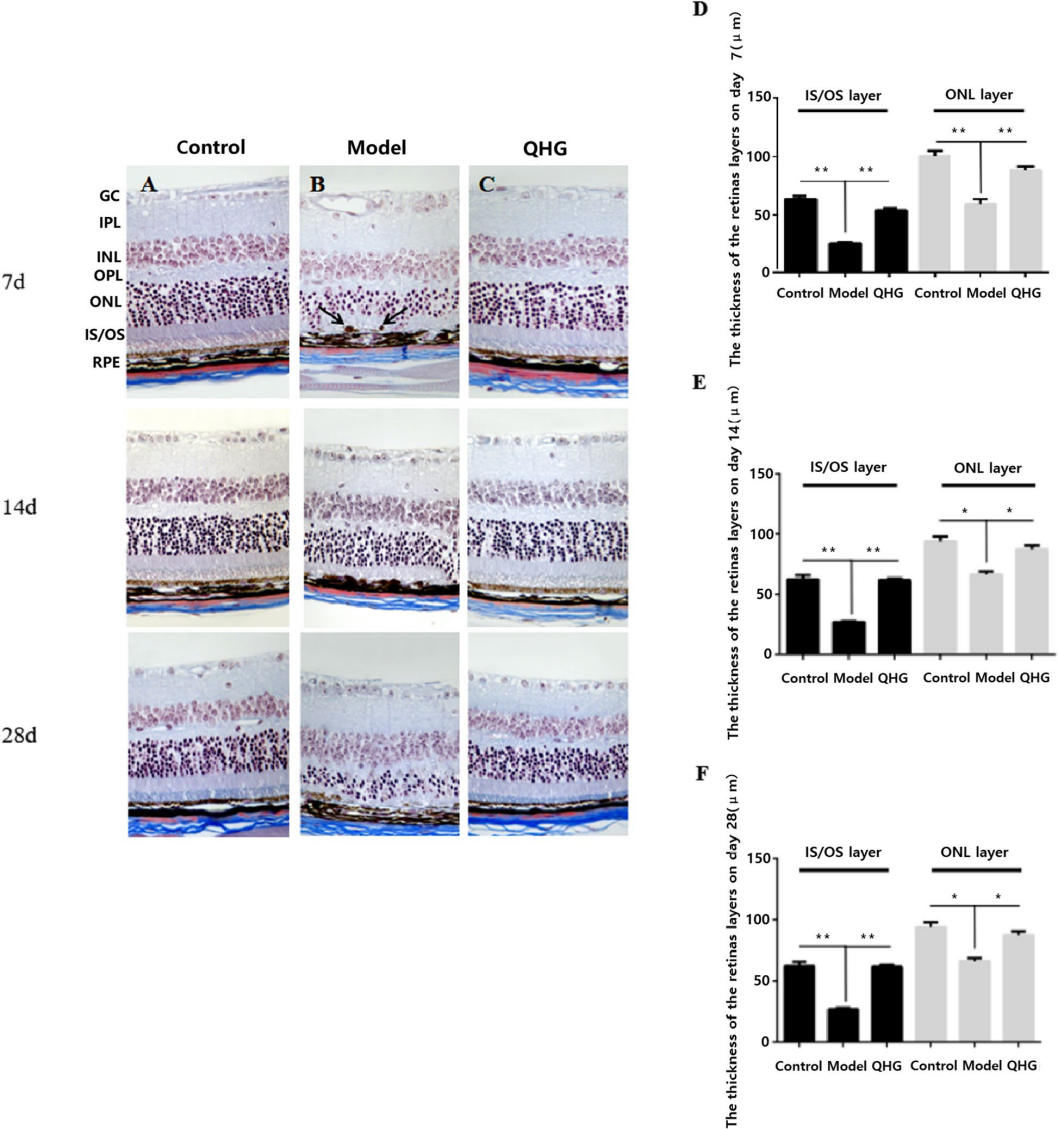
Masson staining of retinal histological sections (**A**-**C**). The black arrow indicates the migration of pigmented cells. Scale bar, 50 µm for all images. GC: ganglion cell, IPL: inner plexiform layer, INL: inner nuclear layer, OPL: outer plexiform layer, ONL: outer nuclear layer, IS/OS: inner segment/outer segment, RPE: retinal pigment epithelium. **D**-**F** Measurement of thicknesses of IS/OS and ONL at 7, 14 and 28 d, respectively. All data are presented as the means ± SDs (*n*=5). *p < 0.05

Histopathological changes in mouse retinas were examined using Masson staining. All layers of a healthy retina were found in the control mouse group (Fig. 2A-C). Retinas in the model group exhibited RPE swelling and migration of pigmented cells into the IS/OS layer. Moreover, the IS/OS and the outer nuclear layer (ONL) were thinner (Fig. 2D-F) than those in the control group. However, the abnormal histopathological changes in the QHG treatment group were significantly ameliorated.

The layer became thicker, and the pigment granules became ordered. The IS/OS and ONL layers were also remarkably thicker at 7, 14 and 28 d (Fig. 2C-F). These findings indicated that QHG protected RPE cells from oxidative stress injury in mice.

### QHG ameliorated NaIO_3_-induced injury to the retinal ultrastructure structure in mice

**Fig. 3:**
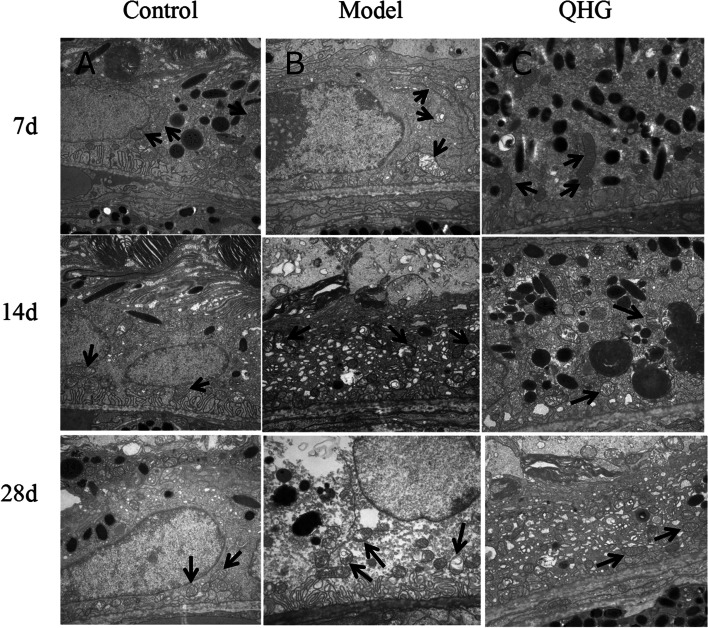
Electron micrograph of RPE cells (magnification 15000×). Top, middle, and bottom: days 7, 14 and 28, respectively. Left to right: control, model, and QHG groups. The black arrow indicates the mitochondria in RPE cells. Scale bar, 1 µm for all images

Retinal ultrastructural changes were detected using TEM. Fig. 3A shows that normal mitochondrial structures were observed in the RPE cells in the control group. However, the model group exhibited swollen mitochondria, cristae fragmentation, granulovacuolar degeneration bodies, and obvious cytoplasmic lysis in RPE cells at 7, 14 and 28 d (Fig. 3B). QHG treatment ameliorated the abnormal morphology of mitochondria and cytoplasmic lysis in RPE cells (Fig 3C). Our findings suggested that QHG protected the mitochondria in RPE cells from oxidative stress damage.

### Oxidative stress upregulated CFH expression and downregulated C3a and C5a expression

**Fig. 4:**
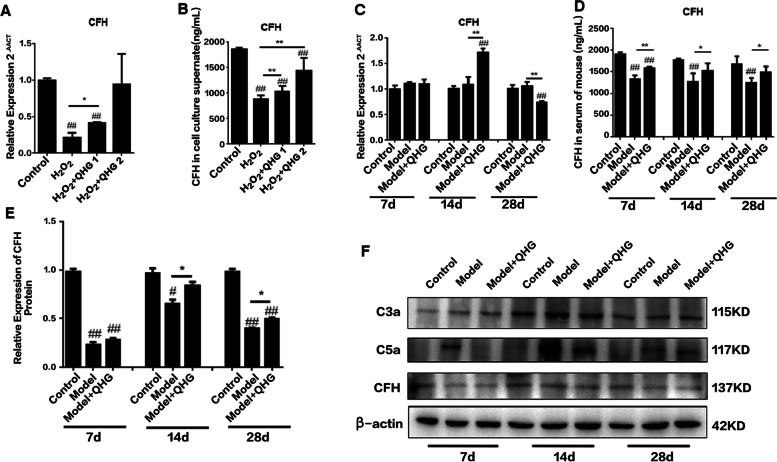
Effects of QHG on CFH expression. **A**. CFH mRNA levels in ARPE-19 cells. **B** CFH protein levels in ARPE-19 cell culture supernatants. **C** CFH mRNA levels in mouse retinas. **D** CFH protein levels in mouse serum. **E**, **F** CFH protein levels in the mouse retina. All data are presented as the means ± SDs (*n*=5). ^*#*^*p < 0.05*, ^*##*^*p < 0.01* vs. Control; *p < 0.05, **p < 0.01 vs. Model

**Fig. 5:**
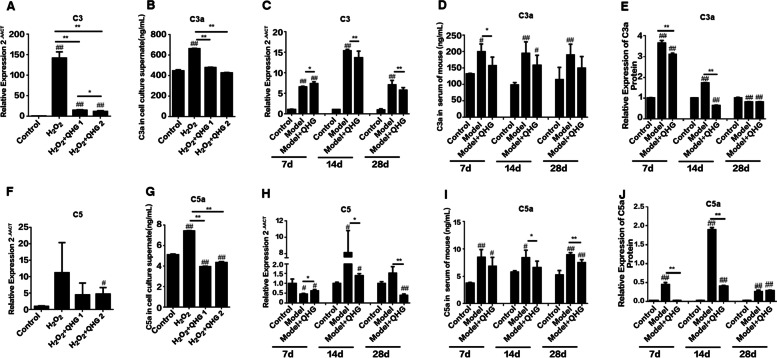
Effects of QHG on C3a and C5a expression. **A**. C3 mRNA levels in ARPE-19 cells. **B** C3a protein levels in the ARPE-19 cell culture supernatant. **C** C3a mRNA levels in the mouse retina. **D** C3a protein levels in mouse serum. **E** C3a protein levels in the mouse retina. **F** C5 mRNA levels in ARPE-19 cells. **G** C5a protein levels in the ARPE-19 cell culture supernatant. **H** C5 mRNA levels in the mouse retina. **I** C5a protein levels in mouse serum. **J** C5a protein levels in the mouse retina. All data are represented as the mean ± SD (*n*=5). ^*#*^*p < 0.05*, ^*##*^*p < 0.01* vs. Control; *p < 0.05, **p < 0.01 vs. Model

Many studies have demonstrated that oxidative stress triggers the activity of the alternative complement system [25], but the definitive interaction between complement factors CFH, C3a and C5a and oxidative stress is not known. We measured the expression of CFH, C3a and C5a under oxidative stress conditions induced by H_2_O_2_ and NaIO_3_. We exposed ARPE-19 cells to 200 μM H_2_O_2_ for 24 h *in vitro* and then measured the mRNA and protein expression of CFH, C3a and C5a. For the *in vivo* study, mice in the model group received NaIO_3_ (40 mg/kg) via the tail vein, and the mRNA and protein expression of CFH, C3a and C5a were measured on days 7, 14 and 28. The level of CFH mRNA was significantly reduced, and the levels of C3a and C5a mRNA were increased *in vivo* and *in vitro* (Figs. 4A, C and 5A, C). Western blotting and ELISA were used to evaluate whether oxidative stress affected protein expression. The results showed that the protein levels of CFH were significantly downregulated compared to the control group (Fig. 4B, D, E), but C3a and C5a expression was remarkably upregulated (*p*<0.05) (Fig. 5B, D-E, G, I-J). These results indicated that oxidative stress caused a dysregulation of CFH, C3a and C5a and induced abnormal activity of the alternative complement system.

### QHG restored the oxidative stress-induced loss of CFH expression in RPE cells and mice

CFH is a vital complement factor and the enzyme responsible for inactivating the alternative complement pathway cascade [26, 27]. We investigated whether QHG regulated CFH expression under oxidative stress *in vitro* and *in vivo*. ARPE-19 cells were pretreated with 1 and 2 mg/ml QHG for 24 h and then exposed to 200 μM H_2_O_2_ for 24 h. Mice in the QHG group were intragastrically administered QHG for 7 d prior to NaIO_3_ injection. After NaIO_3_ injection, the mice were continuously administered QHG for 7, 14 or 28 d. The mRNA and protein expression of CFH were measured. The results demonstrated that treatment with QHG significantly improved the expression of CFH mRNA in RPE cells and mouse retinas (Fig. 4A, C) and upregulated CFH protein expression in ARPE-19 cell culture supernatants, mouse sera and mouse retinas (Fig. 4B, D, E) compared to the model group. These results indicated that QHG restored the oxidative stress-induced loss of CFH in RPE cells and mice.

### QHG decreased the release of C3a and C5a in RPE cells and mice

C3a and C5a are important alternative complement components that act as proinflammatory stimuli [28]. We also tested whether QHG regulated the expression of C3a and C5a under oxidative stress *in vitro* and *in vivo*. The results showed that QHG significantly decreased the H_2_O_2_- and NaIO_3_-induced increases in the mRNA and protein expression of C3a and C5a in ARPE-19 cells and mouse serum and mouse retinas, respectively (Figs. 4F and 5A-J).

## Discussion

Dry AMD is a degenerative disorder of the macula that threatens millions of people worldwide [2]. However, there are no effective drugs to treat this disease [3, 4]. The Age-Related Eye Disease Study trials only suggest the use of daily antioxidant micronutrient supplements for dry AMD patients [29, 30]. Many herbal formulas are popularly used among dry AMD patients in Asian countries [31, 32]. QHG is an herbal formula that is commonly used to treat mild-to-moderate AMD in China. Our previous study demonstrated that QHG improved the vision of AMD patients [17], but its therapeutic mechanism remains elusive.

The RPE is a monolayer of epithelial cells located between the photoreceptors and the choroid, and it is very susceptible to oxidative stress [7]. RPE degeneration plays a central role in dry AMD. Many studies have demonstrated that mitochondrial dysfunction is closely associated with RPE degeneration, and oxidative stress results in morphological and functional changes in mitochondria in RPE cells [9, 36]. Therefore, protection of RPE cells from oxidative stress injury is critical in the treatment of dry AMD. The present study established oxidative stress-induced AMD models using H_2_O_2_ and NaIO_3_. We demonstrated that H_2_O_2_ triggered considerable RPE cell apoptosis and decreased cell metabolic activity. NaIO_3_ caused RPE swelling, disorganization of photoreceptors and significant thinning of the ONL in mice. TEM revealed oedema and the presence of many vacuoles in the mitochondria and high levels of cytoplasmic lysis in RPE cells in the NaIO_3_-induced mice. Our results are consistent with previous reports [34–36]. We used appropriate doses of QHG to pretreat cells and mice. We found that pretreatment with QHG significantly decreased the apoptosis of RPE cells, alleviated the disordered RPE and IS/OS and thickened the ONL in mice. We also showed that the abnormal structure of mitochondria in RPE cells was alleviated in QHG-treated mice. These findings indicate that QHG alleviated cell apoptosis and mitochondrial damage in RPE cells and protected mouse retinas from oxidative stress injury.

Oxidative stress triggers endogenous complement-dependent inflammatory responses and results in local chronic retinal inflammation in dry AMD [25]. Our findings indicated that QHG protected retinas and RPE cells from oxidative damage, but the potential mechanisms were not known. Whether QHG exerts a protective effect on the retina via the alternative complement pathway is not known. To answer these questions, we performed *in vitro* and *in vivo* experiments to examine the interactions between complement factors and oxidative stress and the effects of QHG on the alternative complement system.

CFH, which is derived from the blood circulation and RPE, is the primary regulator of the alternative complement pathway [26, 27]. CFH negatively regulates the alternative complement pathway feedback loop and exerts anti-inflammatory functions by binding to host surfaces to protect against complement activation [7, 14]. We observed the changes in CFH expression during conditions of oxidative damage. We found that the expression of CFH mRNA and protein was reduced in H_2_O_2_-treated RPE cells and NaIO_3_-injected mice. Our results are consistent with previous reports that showed that oxidative stress activated the alternative complement pathway [37, 38]. However, treatment with QHG significantly restored the expression of CFH mRNA and returned the downregulated CFH protein level to normal levels *in vitro* and *in vivo*. Our current study suggests that QHG administration upregulates CFH levels under oxidative stress conditions.

Fragments of C3 and C5, C3a and C5a, act as anaphylatoxins and play a distinct role in the process of AMD [28]. These proteins are critical chemoattractant proteins that promote the recruitment and activation of phagocytic immune cells to sites of tissue damage and the production of proinflammatory cytokines, such as TNF-α and IL-1, which cause local chronic inflammation [28, 39, 40]. Our study revealed that oxidative stress increased C3a and C5a release in RPE cells and mouse retinas, which is consistent with a previous study [27]. This finding confirmed that oxidative stress activated the alternative complement pathway. However, the QHG group exhibited low C3a and C5a expression in RPE cells and mouse retinas. Therefore, QHG decreased the C3a and C5a levels, which may suppress local retinal inflammation. Taken together, our results suggest that QHG has antioxidative and anti-inflammatory effects, and it protects the RPE from oxidative stress via regulation of the alternative complement pathway, which may halt or delay disease progression in AMD.

QHG is a combination of four constituents, and Salvia miltiorrhiza Bunge and Lycium barbarum L. are the main constituents. These two main herbs exhibited antioxidant [41], immunoregulatory [42] and retinal protective effects [41, 43, 44] in previous studies. Prior experiments showed that tanshinone, which is an active component of Salvia miltiorrhiza, regulated the complement cascade pathway in an acute myocardial infarction model in rats [42]. Jian WJ et al. reported that the Fufang Xueshuantong capsule, which contains the main bioactive constituent tanshinone, had retina-protecting effects in diabetic mice [43]. Hsieh et al. reported that Lycium barbarum extracts prevented RPE cell apoptosis and UVB irradiation-induced DNA damage [41]. Zhu Y reported that Lycium barbarum polysaccharides inhibited rat photoreceptor cell apoptosis and protected retinal structure [44]. These studies help us understand our results.

## Conclusion

QHG exhibited a protective role in experimental AMD models. QHG reduced RPE cell apoptosis and repaired oxidative stress-induced mitochondrial and retinal damage. CFH, C3a and C5a are the key proteins of the alternative complement pathway. QHG regulated the expression of key proteins in the alternative complement pathway. This study only revealed one pathway by which QHG acts. Hence, further ascertaining the involvement of other pathways is essential for future studies and drug therapy development in the treatment of AMD.

## Supplementary Information

**Additional file 1.**

## Data Availability

All data included in this study are available upon reasonable request by contact with the corresponding author.

## Abbreviations

AMD: Age-related macular degeneration
C3: Complement 3
C5: Complement 5
C3a: Complement component 3a
C5a: Complement component 5a
CFB: Complement factor B
CFH: Complement factor H
ELISA: Enzyme-linked immunosorbent assay
IS/OS: Inner segment/outer segment
ONL: Outer nuclear layer
QHG: Qihuang Granule
ROS: Reactive oxygen species
RPE: Retinal pigment epithelium
TEM: Transmission electron microscopy
TCM: Traditional Chinese medicine
