# Supplementary figures and images for: Qihuang Granule protects the retinal pigment epithelium from oxidative stress via regulation of the alternative complement pathway
Source: BMC Complement Med Ther. 2023 Feb 18;23:55. doi: 10.1186/s12906-023-03884-2 (PMC9938598; doi:10.1186/s12906-023-03884-2)

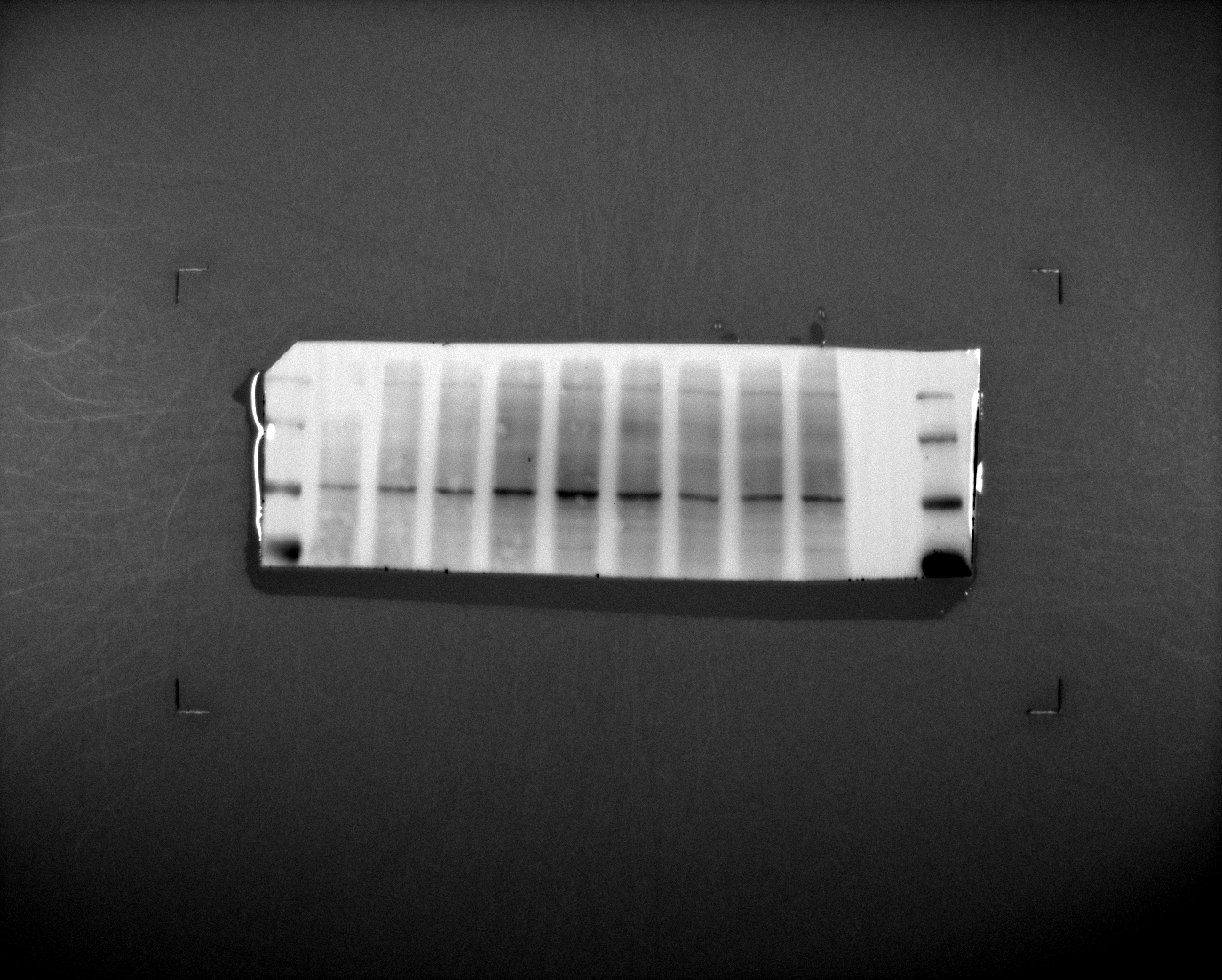

Supplement: Supplementary file 1 — Additional file 1. [file 12906_2023_3884_MOESM1_ESM.zip › C3a.tif]

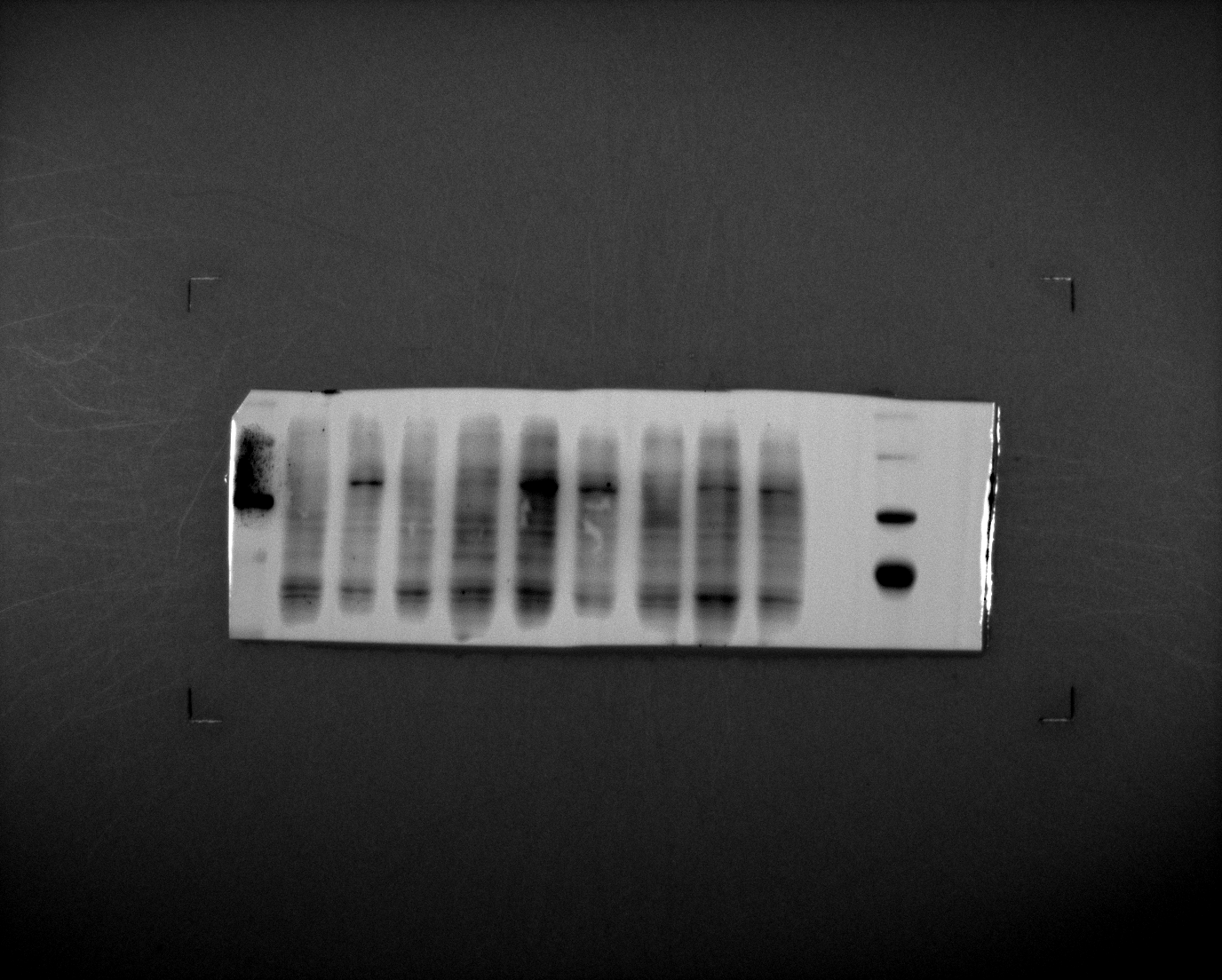

Supplement: Supplementary file 1 — Additional file 1. [file 12906_2023_3884_MOESM1_ESM.zip › C5a.tif]

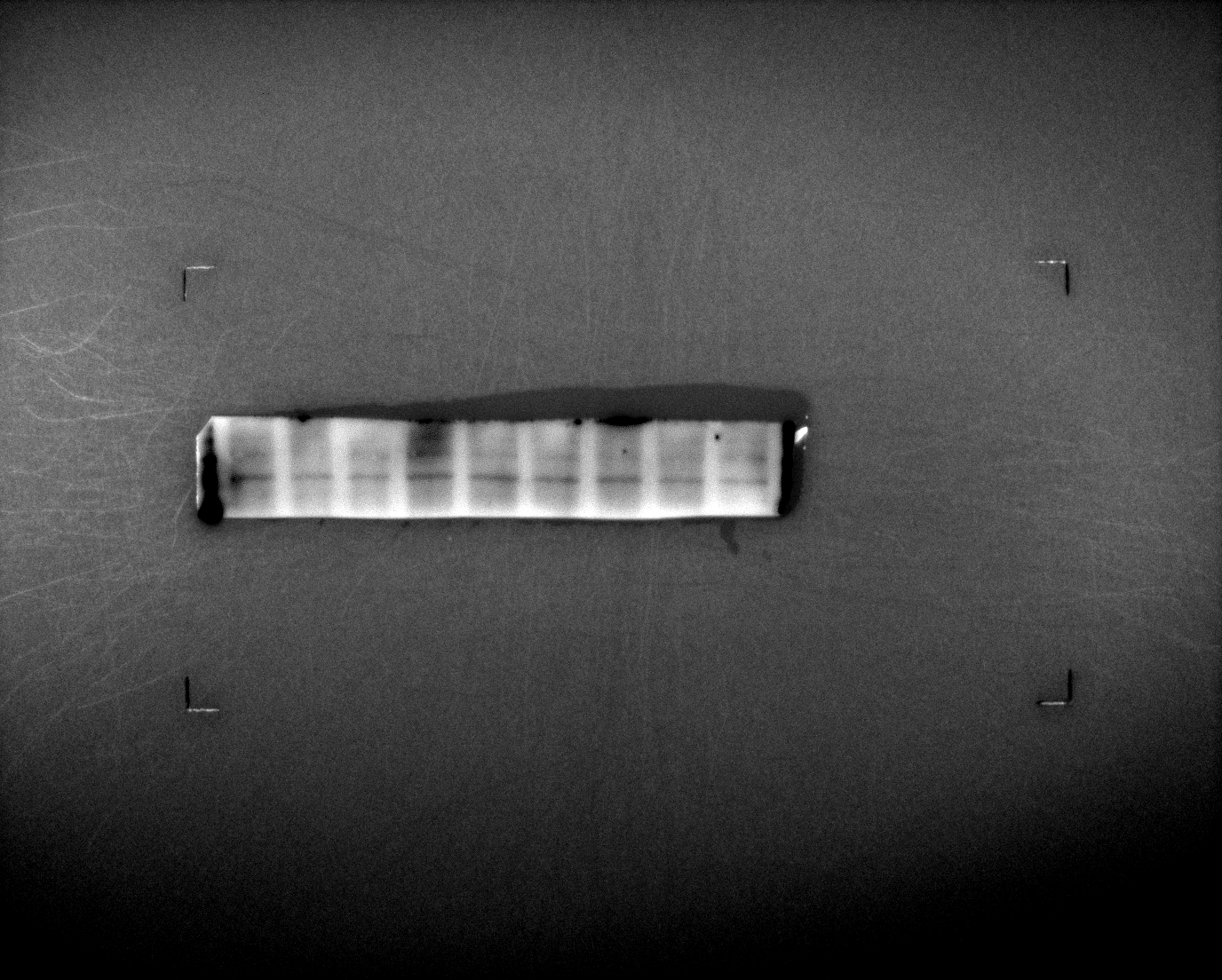

Supplement: Supplementary file 1 — Additional file 1. [file 12906_2023_3884_MOESM1_ESM.zip › CFH.tif]

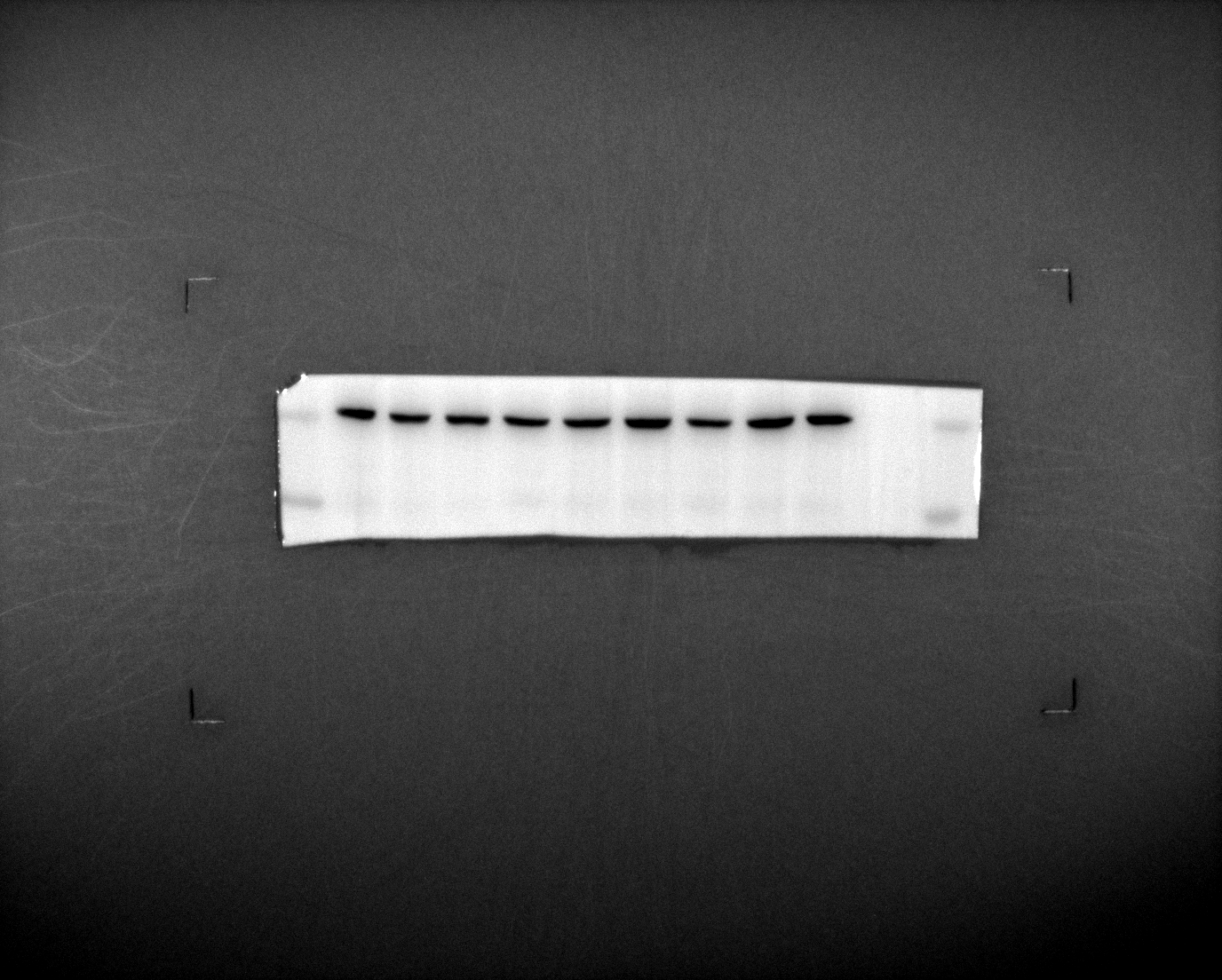

Supplement: Supplementary file 1 — Additional file 1. [file 12906_2023_3884_MOESM1_ESM.zip › ß-actin.tif]
